# The provision of out-of-hours care and associated costs in an urban area of Switzerland: a cost description study

**DOI:** 10.1186/1471-2296-11-99

**Published:** 2010-12-20

**Authors:** Klaus Eichler, Daniel Imhof, Corrine Chmiel Moshinsky, Marco Zoller, Oliver Senn, Thomas Rosemann, Carola A Huber

**Affiliations:** 1 Institute of Health Economics, Zurich University of Applied Sciences, Winterthur, Switzerland; 2 Institute of General Practice and Health Services Research, University of Zurich, Zurich, Switzerland

## Abstract

**Background:**

In Switzerland, General Practitioners (GPs) play an important role for out-of-hours emergency care as one service option beside freely accessible and costly emergency departments of hospitals. The aim of this study was to evaluate the services provided and the economic consequences of a Swiss GP out-of-hours service.

**Methods:**

GPs participating in the out-of-hours service in the city of Zurich collected data on medical problems (ICPC coding), mode of contact, mode of resource use and services provided (time units; diagnostics; treatments). From a health care insurance perspective, we assessed the association between total costs and its two components (basic costs: charges for time units and emergency surcharge; individual costs: charges for clinical examination, diagnostics and treatment in the discretion of the GP).

**Results:**

125 GPs collected data on 685 patient contacts. The most prevalent health problems were of respiratory (24%), musculoskeletal (13%) and digestive origin (12%). Home visits (61%) were the most common contact mode, followed by practice (25%) and telephone contacts (14%). 82% of patients could be treated by ambulatory care. In 20% of patients additional technical diagnostics, most often laboratory tests, were used. The mean total costs for one emergency patient contact were €144 (95%-CI: 137-151). The mode of contact was an important determinant of total costs (mean total costs for home visits: €176 [95%-CI: 168-184]; practice contact: €90 [95%-CI: 84-98]; telephone contact: €48 [95%-CI: 40-55]). Basic costs contributed 83% of total costs for home visits and 70% of total costs for practice contacts. Individual mean costs were similarly low for home visits (€30) and practice contacts (€27). Medical problems had no relevant influence on this cost pattern.

**Conclusions:**

GPs managed most emergency demand in their out-of-hours service by ambulatory care. They applied little diagnostic testing and basic care. Our findings are of relevance for policy makers even from other countries with different pricing policies. Policy makers should be interested in a reimbursement system promoting out-of-hours care run by GPs as one valuable service option.

## Background

Across Europe several reforms of emergency services provided by hospitals or General Practitioner (GP) networks are currently under way. These projects have been evaluated to gain deeper insights into changes of patient care and access to services, as well as into economic effects [1-4].

In Switzerland, GPs provide an out-of-hours emergency service that is an important element in emergency care beyond sporadic urban walk-in emergency centres or costly emergency wards of hospitals. Patients with emergency health problems can call an Emergency Medical Service Telephone and are connected to a GP on duty or an ambulance is sent in case of serious emergency. In addition, there is free access to walk-in emergency centres or hospital emergency departments. As no universal gate-keeping system exists in Switzerland, hospital emergency wards are increasingly used for non-serious health problems that could be treated by GPs. This leads to an inefficient use of public resources.

There has been little Swiss research into the mode of care delivery of the GP out-of-hours service and on relevant factors that affect its total costs [5]. If this emergency service proves to be a valuable service option at reasonable costs, such knowledge might be important for decision makers to allocate scarce health care resources in a rational manner.

Thus, we evaluated a Swiss GP out-of-hours service and assessed the mode of care delivered. In addition, a cost analysis was conducted to better understand the economic consequences of this service.

## Methods

We conducted a cross-sectional questionnaire survey and performed a cost description study [6]. The results of our study, concerning the demand of care, the reasons for encounter and physician satisfaction with this out-of-hours service, have been reported elsewhere [7].

### Setting

The study took place in the city of Zurich (400'000 inhabitants), Switzerland. We covered two time periods (from JAN 1st to FEB 28th, 2009; from AUG 17th to SEP 28th, 2009) to take into account seasonal variability of diseases.

In Zurich, patients with emergency health problems contact an Emergency Medical Service Telephone (EMST) as a unit of the general emergency medical service. The EMST provides a telephone triage and patients are then connected to the physician on duty [7]. The EMST also coordinates GP rota groups and acute mental health care. GPs have to provide a mandatory out-of-hours service from 7 a.m. to 7 a.m. the following day, in a rota system. For each of five service areas one GP is on duty. Between 10 p.m. and 7 a.m. a so called "night physician" of a deputizing service provides out-of-hours care and the GP is on back-up service.

### Subjects and data collection

We included each individual Zurich GP, who performed the traditional mandatory out-of-hours service during the study periods in one of the five services areas, i.e. each day 5 GPs were included. For the period AUG 2009 to SEP 2009 the night physician was also included. We did not obtain formal consent from the GPs, but the EMST coordinated the mailing and motivated them to participate. Walk-in emergency centres were not included. The questionnaire for data collection was mailed to the GP on duty by the EMST and two telephone reminders were used to increase return rate.

The GP collected data for number and mode of contact (home, practice or telephone contact), patient variables (age, gender), medical problems (coded according to International Classification of Primary Care, ICPC-2 [8]), treatment and mode of care delivery after emergency contact (e.g. final treatment by GP defined as ambulatory care or transferral to hospital). Furthermore, the urgency level was categorised as "self care (by patient) sufficient", "medical care indicated" and "medical emergency". In addition, services provided by GPs were documented, e.g. mode and number of time units for consultation or travelling (if applicable), diagnostic procedures and medical treatments. This detailed clinical and economic data was collected for the first, second and last patient of each single participating GP during the 24-hours-service and procured via the EMST.

Approval of the study was given by the local ethics committee (reference Nr. 26/09).

### Perspective of economic evaluation and pricing

In Switzerland, health care insurance is part of the social insurance system and mandatory for all citizens. For the ambulatory sector, as covered by our study, health care insurance companies have to pay the charges of GPs for their services they provide. Thus, our analysis was performed from the perspective of a health care insurance company as the relevant payer. We calculated 2009 prices in Swiss Francs (CHF; with conversion by factor 0.67 to Euros, €) by multiplying the number of resource units and services by unit prices, taking emergency surcharge into account. We used the obligatory Swiss pricing lists for ambulatory care (TARMED, version 1.05.03; Analysenliste, version 1.1.2006 and update [9,10]). We did not consider a patient perspective, as data for out-of-pocket co-payments are not systematically available in Switzerland. The amount of co-payment in Switzerland is among the highest in OECD countries [11] but restricted to 10% of service prices plus a deductible of at least 300 CHF (to a maximum of 700 CHF per year).

### Statistics

For our descriptive analysis, we used means (SD) for continuous variables and proportions for categorical data. For inferential analysis we applied parametric and non-parametric tests.

To assess the economic impact of relevant components of this GP emergency service, we used a stepwise approach.

Firstly, we assigned all cost components to one of two groups. We grouped essential cost components as "basic costs". Such components have fixed prices (e.g. emergency surcharges) or may have limited quantities for charging in Switzerland (e.g number of 5-minute time units for consultation) and are applied to all patients. All other cost components were grouped as "individual costs". These components comprise measures at the discretion of each individual GP and are directly linked to the specific treatment of patients. Examples of components at the GP's discretion are type of clinical examination and counselling or type and number of applied diagnostics and treatments, if any. For individual costs, no maximum number of chargeable services is defined. Basic costs and individual costs add up to total costs.

Secondly, we calculated mean total, mean basic and mean individual costs. For inferential analysis, we calculated 95%-confidence intervals (CI) using the non-parametric bootstrap [12].

Thirdly, we calculated the Spearman's rank correlation coefficient (r_s_) to assess the strength and direction of association between total costs and each of the two cost components. We also did this for the association between basic costs and individual costs. Furthermore, we designed a scatter plot (with total costs on the x-axis and basic costs on the y-axis) to assess the distribution of individual patient data graphically [13].

Data analysis was conducted with SPSS for Windows, version 18.0 (SPSS Inc., Chicago, Illinois); for bootstrapping we used Stata 9.0 (StataCorp 2004, Stata Statistical Software, College Station, TX).

## Results

### Patients, GPs and medical problems

**Figure 1:**
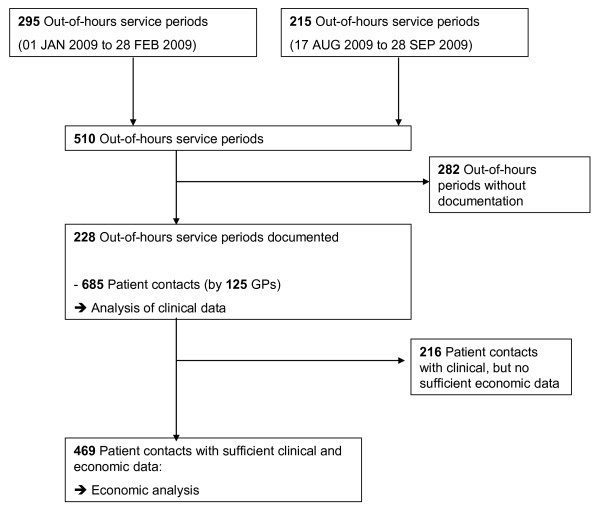
**Study flow**.

The GP response rate in our study was 45% (228 of all 510 out-of-hours service periods were documented) and 125 GPs collected data for 685 patient contacts (Figure 1). The mean patient age was 59 years (Table 1), 65% of the patients were female. The GPs' mean age was 49 years. For 469 of 685 (69%) patient contacts sufficient data were available for economic analysis.

**Table 1:** Patients' and GPs' Characteristics

	General Practitioners*	All patients*	Patients with economic analysis*	Patients without economic analysis*
	**N = 125**	**N = 685**	**N = 469**	**N = 216**

**Gender**^#^	n = 125	n = 323	n = 233	n = 90

Women, No. (%)	31 (24.8)	210 (65.0)	147 (63.1)	63 (70.0)

Men, No. (%)	94 (75.2)	113 (35.0)	86 (36.9)	27 (30.0)

**Age**	n = 124	n = 525	n = 415	n = 110

**Years, mean (SD)**	49 (5.9)	59 (23.8)	60 (24.0)	55 (22.7)

**Experience in the current out-of-hours service**	n = 101			

<2 years, No. (%)	11 (10.9)	-	-	-

2-5 years, No. (%)	15 (14.9)	-	-	-

6-10 years, No. (%)	26 (25.7)	-	-	-

>10 years, No. (%)	49 (48.5)	-	-	-

**GP of their own**		n = 567	n = 455	n = 112

Patient with GP, No. (%)	-	481 (84.8)	385 (84.6)	96 (85.7)

Patient without GP, No. (%)	-	86 (15.2)	70 (15.4)	16 (14.3)

*For each subgroup the number of patients with valid data is indicated; ^#^Patient gender relates to period JAN to FEP 2009;

From the GPs' view, in 27% of cases the urgency of the patient problem was judged as a "medical emergency" (Table 2). The three most prevalent groups of diagnoses were of respiratory (28%; 161 of 582 patients with ICPC-data), musculoskeletal (16%; 92 of 582) and digestive origin (15%; 85 of 582). The four most frequently documented single diagnoses (Influenza n = 45; back syndrome n = 39; upper respiratory tract infection n = 35; gastroenteritis presumed infection n = 29) accounted for 25% of 582 patient contacts with data about diagnoses.

**Table 2:** Mode of contact, medical problems and care delivered

	All patients*	Patients JAN to FEB 2009*	Patients AUG to SEP 2009*	Patients with economic analysis*	Patients without economic analysis*
	N = 685	N = 445	N = 240	N = 469	N = 216
**Mode of contact**	n = 578	n = 380	n = 201	n = 459	n = 119

Practice consultation, No. (%)	147 (25.4)	102 (26.8)	45 (22.4)	118 (25.7)	29 (24.4)

Home visit, No. (%)	351 (60.7)	226 (59.6)	125 (62.2)	302 (65.8)	49 (41.2)

Telephone contact (only), No. (%)	80 (13.9)	52 (13.7)	28 (13.9)	39 (8.5)	41 (34.5)

**Urgency of medical problem**	n = 560	n = 370	n = 190	n = 448	n = 112

Medical emergency (from GP's view), No. (%)	149 (26.6)	96 (25.9)	53 (27.9)	128 (28.6)	21 (9.7)

**Medical problem (ICPC-2 chapter)**	n = 582	n = 383	n = 189	n = 463	n = 119

Digestive problem, No. (%)	85 (14.6)	50 (13.1)	35 (17.6)	70 (15.1)	15 (12.6)

Musculoskeletal problem, No. (%)	92 (15.8)	60 (15.7)	32 (16.1)	78 (16.8)	14 (11.8)

Respiratory problem, No. (%)	161 (27.7)	135 (35.2)	26 (13.1)	118 (25.5)	43 (36.1)

**Mode of care delivered**	n = 566	n = 373	n = 193	n = 453	n = 113

Ambulatory care by GP, No. (%)	466 (82.3)	311 (83.4)	155 (80.3)	373 (82.3)	93 (82.3)

Transferral to specialist or hospital, No. (%)	100 (17.7)	62 (16.6)	38 (19.7)	80 (17.7)	20 (17.7)

**Diagnostics**^#^	n = 579	n = 377	n = 202	n = 461	n = 118

No diagnostics, No. (%)	467 (80.5)	303 (80.4)	164 (80.8)	367 (79.6)	100 (84.7)

Laboratory tests, No. (%)	89 (15.4)	60 (15.9)	29 (14.4)	73 (15.8)	16 (7.4)

x-ray, No. (%)	7 (1.2)	4 (1.1)	3 (1.5)	4 (0.9)	3 (1.4)

ECG, No. (%)	10 (1.7)	5 (1.3)	5 (2.5)	8 (1.7)	2 (0.9)

Ultrasound, No. (%)	3 (0.5)	2 (0.5)	1 (0.4)	2 (0.4)	1 (0.5)

Other diagnostics, No. (%)	16 (2.8)	7 (1.9)	9 (4.5)	14 (3.0)	2 (0.9)

*For each subgroup the number of patients with valid data is indicated; ^#^Several diagnostics were possible

As expected, diagnoses showed some seasonal variability. For example, respiratory infections were more prevalent during the period JAN to FEB (28%; 108 of 383 patients with ICPC data), compared to the period AUG to SEP (10%; 19 of 189 patients).

Beyond a slightly higher rate of home visits (65.8% vs. 60.7%), the distribution of patient variables, medical problems and mode of care of the 469 patients for economic analysis was similar to that of the total population of 685 patients (Table 2). In patients with incomplete economic data telephone contacts were more usual (35%) and urgent medical problems less frequent (10%).

### Mode of care delivery

Home visits were the most common contact mode (60.7% [95%-CI: 56.7-64.7]) compared to practice contacts (25.4% [95%-CI: 21.9-28.9]) and telephone contacts (13.9% [95%-CI: 11.1-16.7]). 82% of patients could be treated by ambulatory care and 18% had to be referred to specialists or hospitals. General and unspecified symptoms (such as fever), as well as cardiovascular and digestive problems, were the most frequent reasons for referral to a hospital.

In about 20% of cases GPs used additional technical diagnostics to supplement information from patient history and clinical examination. Laboratory tests were applied most often (89/685; 15%), other diagnostics were used rarely (e.g. electrocardiogram: 1.7%; x-ray: 1.2%).

### Cost data

**Figure 2:**
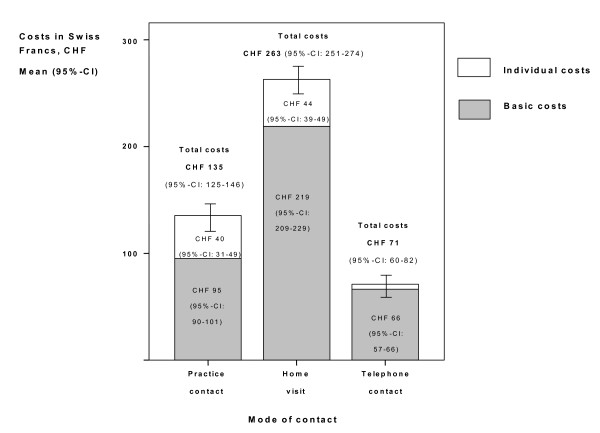
**Costs of emergency care according to mode of contact**. Costs are displayed in Swiss Francs (CHF) according to basic costs (gray) and individual costs (white). Error bars indicate 95%-confindence intervals of total costs for each mode of contact.

The mean total costs (95%-CI) for one emergency patient contact in 469 patients were CHF 215 (205-226) or € 144 (137-151). An important determinant of total costs was the mode of contact. We found significant differences of mean total costs between the different modes of contact (mean total costs [95%-CI] for home visits: CHF 263 [251-274] or € 158 [168-184]; for practice contact: CHF 135 [125-146] or € 90 [84-98]; for telephone contact: CHF 71 [60-82] or € 48 [40-55]; Figure 2) The ranges between the lowest and the highest total costs among all patients with the same contact mode were wide (minimum-maximum range of mean total costs for home visits: CHF 45-872 [€ 30-584]; for practice consultations: CHF 45-347 [€ 30-232]; for telephone contacts: CHF 27-133 [€ 18-89]).

Basic costs (restricted charges for time units and emergency surcharge) averaged 83% (CHF 219 [209-229], € 147 [140-153]) of total costs for home visits and 70% (CHF 95 [90-101], € 64 [60-68]) for practice contacts. Other components added little to the variation in total costs. Individual costs (e.g. for counselling, diagnostics or treatment) were similarly low for home visits (CHF 44 [39-49], € 27 [21-33].

**Figure 3:**
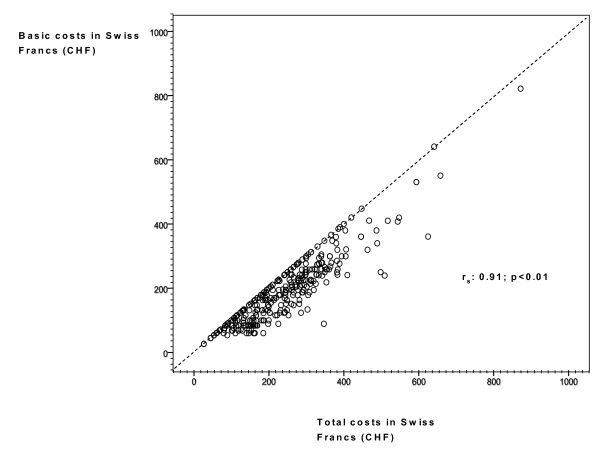
**Scatter plot to assess the association between basic costs and total costs**. The scatter plot shows basic costs on the y-axis and total costs on the x-axis. Each data point represents a single patient (n = 469 patients with complete cost data). There is a strong linear relationship between basic costs and total costs (Spearman's rank correlation coefficient, r**_s_**: 0.91). Data points on the dashed 45° indifference line represent patients, where total costs are fully represented by basic costs.

Among 469 patients with sufficient cost data, the scatter plot showed a strong linear association between basic costs and total cost (correlation coefficient: r_s _0.91; p < 0.01 Figure 3). The association between individual costs and total cost was moderate (r_s _0.51; p < 0.01). Basic and individual costs did not correlate (for home visits: r_s _0.04; p = 0.50; for practice contacts: r_s _-0.06; p = 0.52).

Mean total costs for the most common diagnoses varied between CHF 154 (138-169) or € 103 (92-113) for upper respiratory infections, CHF 227 (197-257) or € 152 (132-172) for neck/back syndrome and CHF 256 (218-295) or € 172 (146-198) for gastrointestinal infections (patients with telephone contact excluded; Table 3). However, the relative contribution of basic costs (range: 79% to 85%) and individual costs (range: 15% to 21%) to total costs in these patients was similar to that of the total study population.

**Table 3:** Cost components and applied diagnostics for 3 common diagnoses.

	Upper respiratory tract infection	Neck/back syndrome	Gastrointestinal infection
	ICPC codes: R74; R75; R76; R77; R78; R80	ICPC codes:L83; L84; L86	ICPC codes: D70; D73
	N = 66	N = 38	N = 33
**Costs**			

Total costs, CHF, Mean (95%-CI); % of total costs	154 (138-169); 100	227 (197-257)*; 100	256 (218-295)^#^; 100

Basic costs, CHF, Mean (95%-CI); % of total costs	122 (109-135); 79	194 (167-222)*; 85	203 (167-239)^#^; 79

Individual costs, CHF, Mean (95%-CI); % of total costs	32 (24-40); 21	33 (21-45)*; 15	53 (39-68)^#^; 21

**Diagnostics **^†^			

Laboratory tests, No (%)	26 (39)	0 (0)	9 (27)

Other tests, No (%)	2 (3)	0 (0)	2 (6)

No additional diagnostics, No (%)	39 (59)	38 (100)	23 (70)

Cost components in Swiss Francs (CHF) are shown for the three most frequent diagnoses according to ICPC-coding (137 patients treated via "home visit" or "practice contact" and with complete cost data are included, comprising 20% of all 685 patients).

P-values for comparison to costs of patients with upper respiratory tract infections (adjusted for mode of contact): *Neck/back syndrome: total costs p = 0.08; basic costs p = 0.03; individual costs p = 0.91;

^#^Gastrointestinal infections: total costs p < 0.01; basic costs p < 0.01; individual costs p = 0.01;

^†^Subgroups may comprise more than all cases of the group;

## Discussion

Most of the patients seen by GPs during the out-of-hours service were treated by ambulatory care with little diagnostic testing. We found significant differences in total costs depending on the mode of contact. Home visit were the most frequent and the most costly contact mode (CHF 263 or € 158 per contact). Basic costs (restricted charges for time units and emergency surcharge) were the most important cost component and strongly associated with total costs. Individual costs (for clinical examination, diagnostics, treatment) were similarly low for all contact modes and contributed little to total costs.

### Other cost data

Several European studies from the Netherlands and the UK have addressed economic aspects of out-of-hours services [14-16]. However, comparison between different settings with specific pricing policies is difficult and studies applied different costing models or perspectives, compared to our study.

In Switzerland, few economic statistics have been published for out-of-hours services provided by GPs. In these studies, mean prices (revenues based on Tarmed to be paid by healthcare insurance companies) range from CHF 147 to CHF 174 for one out-of-hours patient contact [17,18]. However, studies included only a limited number of patients [18] or prices are no longer up-to-date [17] and no information is given about the modes of contact. In contrast, we were able to assess different factors that affect total costs, such as mode of contact as well as basic and individual costs. Charges for basic care (e.g. for consultation time) have the biggest economic impact on this Swiss out-of-hours service. Basic costs show a strong linear association with total costs and are the determining cost component. This relationship holds in nearly all patients as shown with the scatter plot. In addition, our results showed that basic and individual costs do not correlate. Thus, higher basic costs are not systematically associated with higher individual costs and GPs seem to manage emergency health problems in different situations with a limited number of services. Furthermore, the lack of a negative association between basic and individual costs indicates that GPs do not compensate for lower basic costs with increased individual costs which are at their discretion (e.g. additional technical diagnostics or drugs).

### Strengths and limitations of our approach

Our results contribute to the knowledge base of health services research in emergency care, which is an important field of change for health services across Europe. The study was conducted under the conditions of routine out-of-hours practice of GPs. The pattern of medical problems in our study is comparable to findings of GP emergency care in other countries [14,16,19], which may strengthen the generalisability of our results. Our study was done in an urban area. We believe that the GP out-of-hours service mix for our urban sample (i.e. little diagnostic testing and basic care) applies to rural areas at least as well.

Our study has some limitations. Firstly, transferability of cost data to other countries may be limited, as we applied prices that were negotiated in the Swiss context. However, Swiss GPs applied few simple diagnostic tests and provided basic care to manage patient problems. We believe that prices for such measures are similarly low in other countries. Secondly, selection bias can not be completely excluded, as in only 69% of documented out-of-hours contacts were sufficient economic data available. On the other hand, relevant characteristics of our sub-sample for economic analysis are similar to that of the full study population. Finally, we may have overestimated the costs for Swiss health care insurance companies for out-of-hours emergency ambulatory care, as we could not account for the co-payments of patients. In addition, we may have overestimated the average costs of telephone contacts. One third of telephone contacts showed incomplete economic data and more comprehensive telephone contacts may be overrepresented in the economic analysis.

### Significance of findings and policy implications

Our findings are of significance for decision makers beyond Switzerland. During out-of-hours service, GPs can treat patients with emergent health problems, in home visits as well as in practice contacts, using few diagnostic tests. Costs are mainly determined by basic care. Assuming that GP behaviour is roughly constant across countries, this is relevant information for family-doctor-based out-of-hours models, such as in Australia or Norway [3].

Furthermore, it may have serious cost implications for payers, if emergency patients, who are now seen at home by GPs, are treated in costly hospital emergency wards, some of them possibly transferred via ambulance transport [18,20]. This is an important issue for allocation of scarce health care resources in countries that also do not have an established gate-keeping system for overcrowded emergency departments, such as Germany, Belgium [3] or the US [21].

The relatively high rate of home visits may be seen as potentially costly compared to telephone contacts only. The Swiss profile of out-of-hours care is different from countries, where telephone-management is more usual and home visits are less frequent, such as in the UK or the Netherlands [2]. From the patient perspective, however, there seems to be a certain need for home visits for out-of-hours services in Switzerland [7] and patients may be dissatisfied with services if only telephone advice is given, as shown in the Netherlands [2]. One can presume that our patients, who needed a GP home visit, had a relevant health problem as they had passed the triage of the EMST. Furthermore, they could have made use of freely accessible emergency departments, instead, if they felt healthy enough to leave home.

Satisfaction of Swiss GPs with out-of-hours care is low and recruitment for this service becomes increasingly difficult [22]. Other countries with rota group systems have similar problems [3]. One of the most frequent reasons for dissatisfaction is the view among Swiss GPs that the reimbursement for this service is not sufficient [7]. Our data have shown that GPs can treat ambulatory patients with emergency health problems using low cost measures. Thus, our findings can contribute to future negotiations between policy makers and GPs for fair prices, even in other countries with different pricing policies. This may increase job satisfaction and motivation for recruitment to the GP out-of-hours services.

### Implications for health services research

In many countries, health services research in emergency care has to take into account diverse emergency care models [3]. They contribute to a comprehensive community supply, as the patient spectrum may differ between models [19]. Comparison of patient management, access to services, resource use and costs for complementary emergency services in the same area, provided by (1) hospital emergency departments, (2) primary care centres integrated in hospitals' emergency departments and (3) GPs' out-of-hour-service, can contribute valuable information to decision makers for service planning [23,24]. In this context, specific efforts should be made to account for possible case mix differences, e.g. by application of ICPC-coding to account for variability in medical problems.

Currently, these questions are under study in Zurich [4,7] and results will provide further insights into the economic consequences of different service paths in emergency care. Such data may contribute to the knowledge base of the recently founded European research network for out-of-hours primary health care (EurOOHnet) [25] to better understand the contribution of GPs' out-of-hours service to community emergency systems.

## Conclusions

GPs managed most emergency demand in their out-of-hours service by ambulatory care and applied low cost measures. Thus, policy makers should be interested in a reimbursement system promoting out-of-hours care run by GPs as one valuable service option.

## Competing interests

The authors declare that they have no competing interests.

## Authors' contributions

KE participated in the design of the study and its coordination, performed the statistical analysis and drafted the manuscript. DI participated in the design of the study, and the statistical analysis. CCM, MZ, OS, TR and CAH participated in the design of the study and its coordination, collected data and participated in the statistical analysis. All authors revised the draft manuscript critically for important content and read and approved the final version.

## Pre-publication history

The pre-publication history for this paper can be accessed here:

http://www.biomedcentral.com/1471-2296/11/99/prepub
